# Incidental Discovery of Multiorgan Extramedullary Plasmacytomas in the Setting of Newly Diagnosed Multiple Myeloma and Delayed Hemolytic Transfusion Reaction

**DOI:** 10.1155/2017/4531858

**Published:** 2017-07-06

**Authors:** Joselle Cook, Steven Song, Anthony Ventimiglia, Carol Luhrs

**Affiliations:** ^1^Department of Medicine, SUNY Downstate Medical Center, Brooklyn, NY 11203, USA; ^2^College of Medicine, SUNY Downstate Medical Center, Brooklyn, NY 11203, USA; ^3^Division of Hematology and Oncology, SUNY Downstate Medical Center, Brooklyn, NY 11203, USA

## Abstract

Extramedullary plasmacytomas (EMPs) are defined by the presence of clonal plasma cell proliferation outside of the bone marrow, portending an overall poor prognosis. This case highlights extramedullary plasmacytomas as an unusual presenting manifestation of multiple myeloma. Through incidental discovery during a delayed hemolytic transfusion reaction workup, EMPs were found in the liver, spleen, and possibly the lung. Though rare at presentation, this case emphasizes that the presence of EMPs should be considered at the outset as it not only impacts the treatment regimen for such patients but also considerably affects prognosis.

## 1. Introduction

Multiple myeloma (MM) is the malignant proliferation of plasma cells. It usually occurs in the seventh or eighth decade of life. Newly diagnosed cases often present with symptoms of anemia (fatigue, shortness of breath, and decreased exercise tolerance) and may require RBC transfusions. Through incidental discovery during evaluation of a delayed hemolytic transfusion reaction, multiorgan extramedullary plasmacytomas were found in the liver and spleen, with possible involvement of the lung. This case report reinforces the need for increased awareness of the varied presentations of advanced multiple myeloma.

## 2. Case Presentation

A 58-year-old woman, who recently arrived from Guyana, presented to the primary care clinic with complaints of generalized fatigue, hip pain, and low back pain for the preceding 8 months. She denied fever, chills, unintentional weight loss, abdominal pain, diarrhea, constipation, and cardiopulmonary or neurological symptoms. Routine lab work demonstrated anemia (hemoglobin: 8 mg/dL), hypercalcemia (13.8 mg/dL), and renal impairment (creatinine: 2.83 mg/dL). Remaining labs including liver function tests were remarkable for total protein of 13.4 g/dL. A metastatic bone survey revealed diffuse lytic lesions consistent with multiple myeloma. The patient was admitted to the hospital for management of symptomatic anemia and hypercalcemia and to complete myeloma evaluation.

Intravenous (IV) fluids and dexamethasone were initiated. Further evaluation of the myeloma revealed a monoclonal IgG lambda of 7600 mg/dL and a kappa/lambda ratio of 0.01. Bone marrow biopsy demonstrated 58% plasma cell infiltration. Flow cytometry, immunohistochemistry, and FISH of the biopsy showed a monoclonal IgG-*λ* plasma cell population, with trisomy 11 and 17p del. Spine MRI demonstrated multifocal areas of pathologic marrow replacement of the entire axial spine without signs of cord compression.

**Figure 1:**
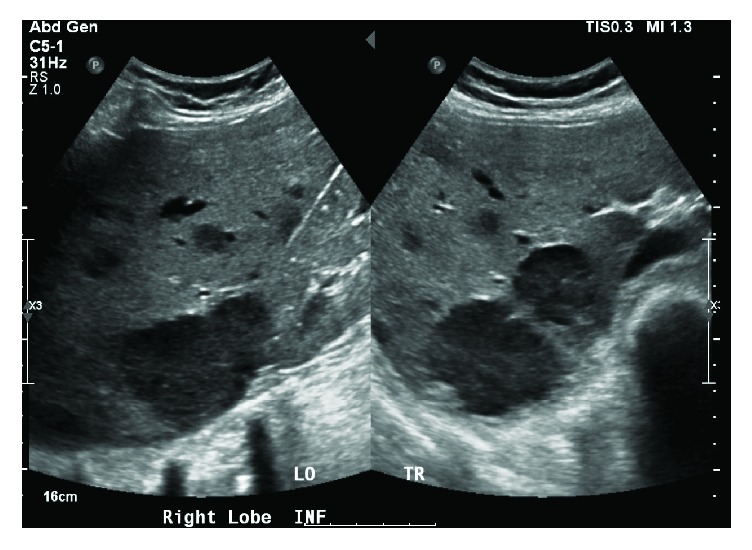
Right lobe liver ultrasound (inferior view) showing innumerable hypoechoic heterogeneous masses, the largest of which was located inferiorly and measured 6.0 × 4.3 × 5.4 cm.

**Figure 2:**
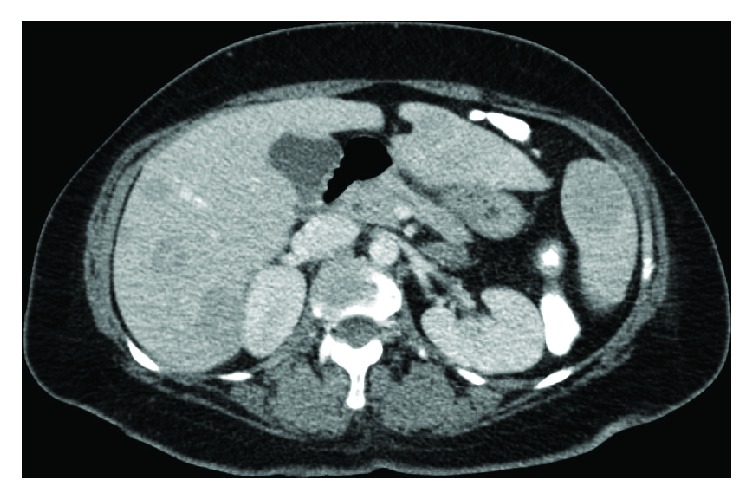
CT of the abdomen showing numerous low attenuation lesions in the liver and spleen suspicious for metastatic disease along with soft tissue nodules in the anterior abdominal wall and numerous lytic lesions in the axial skeleton.

**Figure 3:**
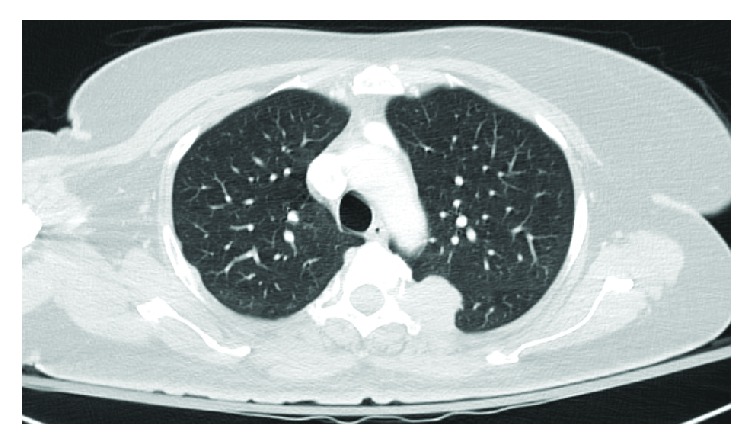
CT of the chest showing a subpleural mass in the upper left lobe that measured 3.3 × 3.0 × 2.7 cm concerning for metastasis.

**Figure 4:**
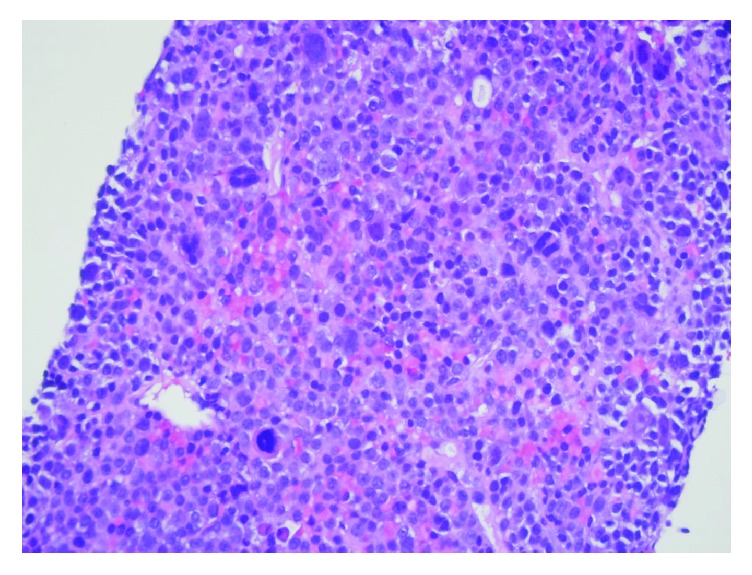
Liver biopsy H&E stain demonstrating compact sheets of atypical plasma cells, consistent with extramedullary hepatic plasmacytoma.

**Figure 5:**
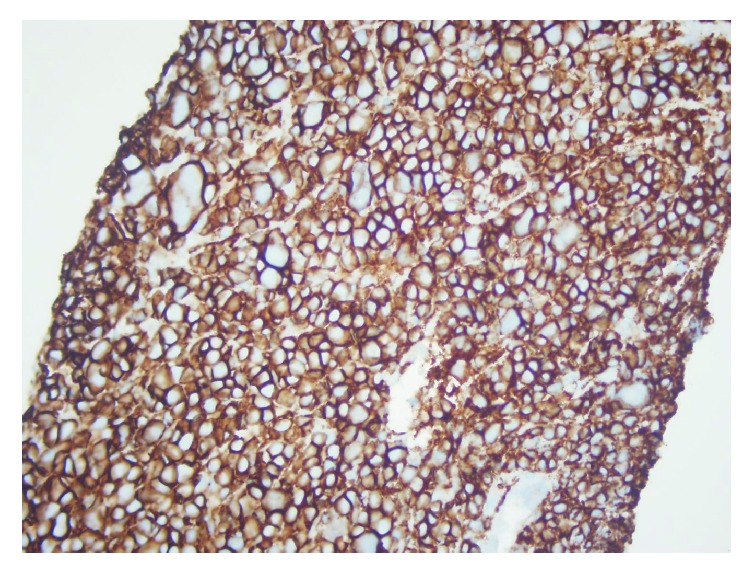
Liver biopsy CD138 marker confirming neoplastic extramedullary plasma cell infiltration from multiple myeloma.

**Figure 6:**
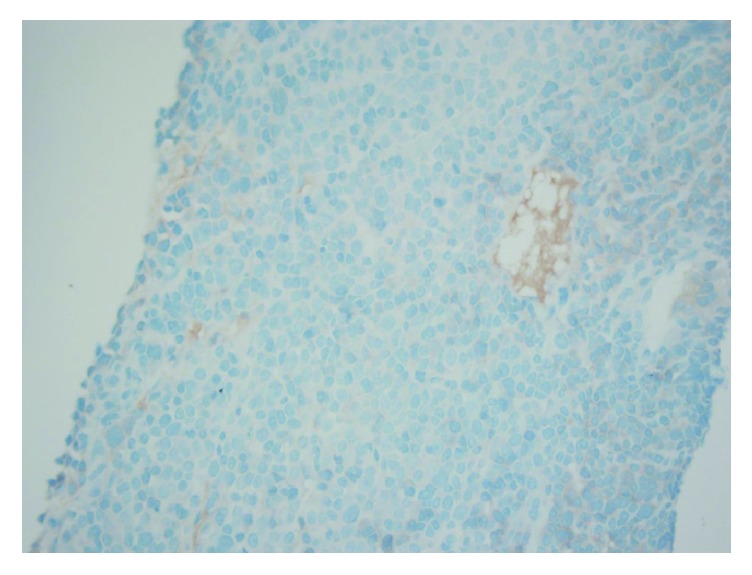
Liver biopsy stained for kappa light chain immunoglobulin confirming neoplastic extramedullary plasma cell infiltration from multiple myeloma.

**Figure 7:**
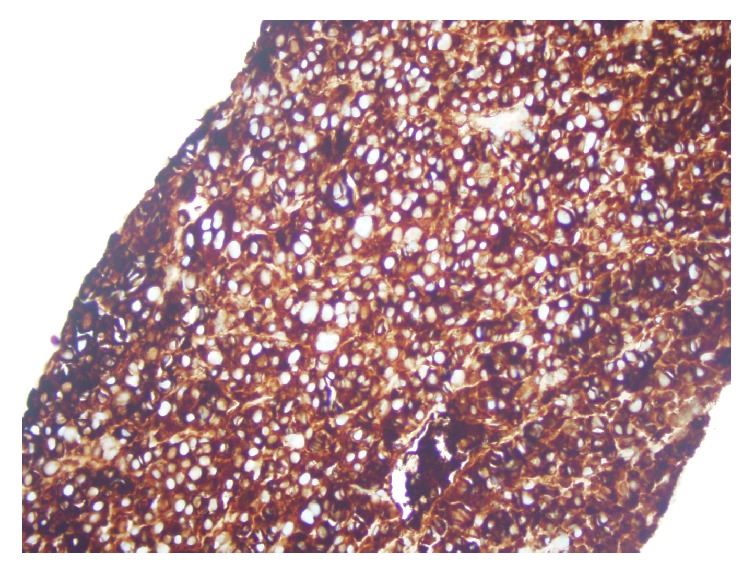
Liver biopsy stained for lambda light chain immunoglobulin confirming neoplastic extramedullary plasma cell infiltration from multiple myeloma.

The patient received one cycle of bortezomib and RBC transfusion for symptomatic anemia. Because of her high risk for pathologic fracture, the patient underwent prophylactic intramedullary nailing of the left humerus and femur. While awaiting right lower extremity intramedullary nailing, there was an acute and unexpected increase in total bilirubin to 4.5 mg/dL, with an indirect predominance and an acute drop in hemoglobin from 8 mg/dL to 6 mg/dL. Other labs were remarkable for elevated LDH (2009 mg/dL), reticulocytosis (absolute reticulocyte count: 354,576 cells/mm^3^), and a positive direct antiglobulin test suggesting a delayed hemolytic transfusion reaction. Right upper quadrant ultrasound performed revealed innumerable hypoechoic hepatic masses (Figure 1). CT of the chest and abdomen showed numerous low attenuation lesions in the liver and spleen suspicious for metastatic disease, along with soft tissue nodules in the anterior abdominal wall and a subpleural mass in the left upper lobe (Figures 2 and 3). Biopsy of one of the hepatic lesions was consistent with extramedullary plasmacytoma (Figures 4–7). After 4 days of supportive care, the hemolysis resolved. Prophylactic right humerus and femur intramedullary nailing was performed, with subsequent initiation of VD-PACE chemotherapy.

## 3. Discussion

Multiple myeloma (MM) is the clonal proliferation of plasma cells originating in the bone marrow that predominantly affects the axial skeleton [1]. Extramedullary plasmacytoma (EMP) is defined by the presence of clonal plasma cell proliferation outside of the bone marrow. Formerly thought to be an uncommon manifestation, EMPs are increasingly being recognized as part of the clinical constellation of MM [2]. The utilization of more sensitive imaging modalities has resulted in earlier recognition of EMPs [3]. The incidence of EMP on diagnosis ranges from 7 to 18%, with a notable association with IgD myeloma [2–4]. EMP is associated with a more aggressive albeit heterogenous natural history, dictated by biologics and genetic mutations unique to this disease subtype [2, 3, 5]. Isolated case reports detail the presence of EMP with an uncharacteristically asymptomatic systemic myelomatous disease [5].

A complexity of high risk cytogenetics and altered tumor biologics within the bone microenvironment signal the aberrant spread of the myeloma cells into the extramedullary realm [2, 5, 6]. Increased expression of CD44, together with low CD56 expression and low p-selectin levels, is a proposed mechanism for EMPs, resulting in decreased adhesion molecule expression and the potential for extramedullary spread [6, 7]. CXCR4 and its ligand CXCL12, thought to mediate the homing of bone marrow progenitor cells, are found to have increased expression with EMPs through induction of an epithelial-mesenchymal transition-like pattern [6, 8]. Increased genetic instability and aberrant cell proliferation have been demonstrated by a high level of mutations in tumor protein p53, high MIB-1 antibody proliferation indices [2, 5, 6]. A demonstrable association has been made between EMPs and poor prognostic cytogenetics such as t[4;14] translocation, 17p deletion, and t[14;16] translocation [2, 6, 9]. As a result of these genetic and cellular irregularities, together with hypoxia and increased angiogenesis, a “bone marrow escape” mechanism is proposed, through extension from skeletal plasmacytomas, or by hematogenous spread to various organs, most commonly lymph nodes, the liver, lung, muscles, the mesentery, and also the skin [2, 5, 7]. Alternatively proposed is the release of plasma cells incited by any surgical intervention during the course of disease [2, 5, 9]. The resultant plasma cells of EMPs are notably less differentiated, with production of predominantly light chains instead of intact immunoglobulins [2, 6]. Patients usually present with more severe manifestations of the myeloma spectrum and high LDH levels [6, 9].

In the diagnostic workup, PET-CT scan would best determine the presence of extramedullary disease and should be performed early as part of initial staging in determined high risk patients [7, 10]. Once the presence of EMPs is established, after the initial PET-CT, it should be repeated to assess response 2 cycles after treatment and at treatment completion to ensure complete remission [2]. It is noteworthy to add that the International Myeloma Working Group recently published a consensus statement recommending the use of PET-CT in the initial workup of all newly diagnosed patients with multiple myeloma, as well as patients with refractory or relapsed myeloma, given its high sensitivity in detecting extramedullary disease as well as its superiority in determining metabolically active disease and early determination of treatment response [11].

With currently evolving practice patterns for MM presenting with EMPs, a favorable response has been seen with the use of high dose combination regimens with lenalidomide, glucocorticoids, chemotherapy with bortezomib, heralded as an “antimyeloma agent,” and cyclophosphamide [2–4, 12]. Improved sustained responses may be seen with high dose therapy and autologous stem cell transplantation after induction therapy in eligible candidates [2–4, 7, 12]. Contrary to previous concerns, several meta-analyses have demonstrated no association between the use of novel agents and targeted treatments such as bortezomib and thalidomide and relapse of extramedullary disease [2, 3, 6, 13].

Outcomes for patients with EMPs at diagnosis continue to emerge through reports of various case series and clinical trials. Varettoni et al. established shorter progression-free survival (PFS) with no significant difference in median overall survival (OS) in patients with EMPs compared to patients without EMPs [3, 6, 7]. Alternatively, outcomes from an autologous stem cell transplant (ASCT) trial by PETHEMA group showed decreased OS, but no difference in PFS [7, 12]. Notably, should relapse of extramedullary disease occur posttreatment, the prognosis for overall survival is significantly worse compared to myeloma without EMPs [6].

In summary, the presence of EMPs is a poor prognosticator, demonstrating decreased overall patient survival, increased rates of disease progression, associated with more severe cytopenias, increased cytogenetic abnormalities, and poorer response to traditional chemotherapeutic regimens [1–3, 9]. This unusual case describes the incidental discovery of extensive EMPs during evaluation of a hemolytic transfusion reaction on the grounds of newly diagnosed multiple myeloma. Myeloma with extramedullary disease proves at this time to be an enigmatic condition with an ominous clinical course, for which definitive treatment and practice patterns are still evolving [5, 6]. High risk genomics and tumor biologics potentially may predict those patients at higher risk of extramedullary disease and should be considered in such patients with newly diagnosed MM [6, 9, 10]. The judicious use of imaging modalities such as PET-CT scan in high risk patients at the initial diagnostic evaluation may facilitate an earlier diagnosis of EMPs.
